# αB-crystallin/HspB5 regulates endothelial–leukocyte interactions by enhancing NF-κB-induced up-regulation of adhesion molecules ICAM-1, VCAM-1 and E-selectin

**DOI:** 10.1007/s10456-013-9367-4

**Published:** 2013-08-09

**Authors:** Lothar C. Dieterich, Hua Huang, Sara Massena, Nikola Golenhofen, Mia Phillipson, Anna Dimberg

**Affiliations:** 1 The Rudbeck Laboratory, Department of Immunology, Genetics and Pathology, Uppsala University, 751 85 Uppsala, Sweden; 2 Department of Medical Cell Biology, Uppsala University, 751 23 Uppsala, Sweden; 3 Institute of Anatomy and Cell Biology, University of Ulm, Albert-Einstein-Allee 11, 89081 Ulm, Germany; 4 Present Address: Institute of Pharmaceutical Sciences, Swiss Federal Institute of Technology (ETH) Zurich, 8093 Zurich, Switzerland

**Keywords:** αB-crystallin, Chaperone, ICAM-1, VCAM-1, E-selectin, NF-κB

## Abstract

αB-crystallin is a small heat shock protein, which has pro-angiogenic properties by increasing survival of endothelial cells and secretion of vascular endothelial growth factor A. Here we demonstrate an additional role of αB-crystallin in regulating vascular function, through enhancing tumor necrosis factor α (TNF-α) induced expression of endothelial adhesion molecules involved in leukocyte recruitment. Ectopic expression of αB-crystallin in endothelial cells increases the level of E-selectin expression in response to TNF-α, and enhances leukocyte–endothelial interaction in vitro. Conversely, TNF-α-induced expression of intercellular adhesion molecule 1, vascular cell adhesion molecule 1 and E-selectin is markedly inhibited in endothelial cells isolated from αB-crystallin-deficient mice. This is associated with elevated levels of IκB in αB-crystallin deficient cells and incomplete degradation upon TNF-α stimulation. Consistent with this, endothelial adhesion molecule expression is reduced in inflamed vessels of αB-crystallin deficient mice, and leukocyte rolling velocity is increased. Our data identify αB-crystallin as a new regulator of leukocyte recruitment, by enhancing pro-inflammatory nuclear factor κ B-signaling and endothelial adhesion molecule expression during endothelial activation.

**Electronic supplementary material:**

The online version of this article (doi:10.1007/s10456-013-9367-4) contains supplementary material, which is available to authorized users.

## Introduction

αB-crystallin (HspB5) is a member of the small heat shock protein family, which is ubiquitously expressed in various cell types and tissues, including endothelial cells, and which can be further induced in response to stress [1, 2]. Through its chaperone function, αB-crystallin affects diverse cellular processes including cytoskeletal rearrangement, production of reactive oxygen species, proliferation and cell survival (reviewed in [3, 4]). Recently, αB-crystallin has emerged as an important regulator of angiogenesis. We have shown that αB-crystallin is up-regulated in endothelial cells during angiogenesis and protects endothelial cells from apoptosis by inhibiting activation of pro-caspase-3 [1]. Consequently, tumor angiogenesis is significantly reduced in *cryab* -*/*- mice, the vessels are hyper-permeable and frequently show signs of endothelial apoptosis. Additionally, αB-crystallin has also been implicated in regulation of physiological and pathological angiogenesis by stabilizing and promoting secretion of vascular endothelial growth factor A (VEGF-A) [5, 6].

Interestingly, we have recently demonstrated a role for αB-crystallin in regulating chronic inflammatory processes, suggesting an additional path through which αB-crystallin expression may affect angiogenesis during pathological conditions. We found that αB-crystallin is expressed in spleen derived, immature CD11b^+^Gr-1^+^ myeloid cells (IMCs), whereas expression in other leukocyte subsets, including mature granulocytes, is negligible [7]. We found that accumulation of IMCs is increased in tumor bearing *cryab* -*/*- mice due to an IMC-intrinsic effect of αB-crystallin. However, a potential contribution of endothelial αB-crystallin to this phenotype has not been investigated. A central step in the inflammatory process is the activation of endothelial cells that mediate recruitment of leukocytes from the blood stream into the inflamed tissue. Pro-inflammatory cytokines such as tumor necrosis factor α (TNF-α) are produced by tissue resident immune cells, inducing activation of local endothelial cells through the nuclear factor κ B (NF-κB) pathway, which in endothelial cells regulates the expression of various chemokines and adhesion molecules such as E-selectin, intercellular adhesion molecule 1 (ICAM-1), and vascular cell adhesion molecule 1 (VCAM-1) [8]. Together, these proteins mediate the capture and adhesion of leukocytes in the blood stream in a well-known multistep process (reviewed in [9, 10]).

In this report, we have employed in vitro models of endothelial activation and intravital microscopy of inflamed vessels to investigate the role of αB-crystallin in regulating inflammatory activation of endothelial cells. Our data demonstrates that αB-crystallin has an important function during this process by enhancing TNF-α-induced activation of NF-κB-signalling and downstream activation of endothelial adhesion molecules.

## Materials and methods

### Mice

Mice deficient for αB-crystallin and HspB2 (*cryab* -*/*-) were described previously [11]. 129S6/SvEvTac wild type mice were obtained from Taconic M&B (Bomholt, Denmark). All animal work was performed according to the guidelines for animal experimentation and welfare provided by Uppsala University and approved by a regional ethics committee.

### Cells

Human embryonic kidney (HEK) 293 T cells were obtained from ATCC (Manassas, VA) and maintained in DMEM (Life Technologies, Carlsbad, CA) + 10 % FCS (Sigma-Aldrich, St. Louis, MO).

Jurkat cells were from ATCC and were cultured in RPMI medium (Life Technologies) + sodium pyruvate (Life Technologies) and 10 % FCS.

Human Umbilical Vein Endothelial Cells (HUVEC) and Human Dermal Microvascular Cells (HDMEC) were from 3H Biomedicals (Uppsala, Sweden) and were maintained on gelatinized culture dishes in endothelial basal medium (EBM-MV2, PromoCell, Heidelberg, Germany) with full supplements. Primary cells were used until passage 10.

MyEnd cells have been described previously [2] and were cultured in DMEM + 10 % FCS.

### Production of lentivirus and transduction of HUVEC

For synthesis of the lentivirus vector *pgk:cryab*, a full length clone of human *cryab* cDNA (NM_001885.1, Origene, Rockville, MD) was cloned into a modified lentiviral *pgk* vector [12] containing an internal ribosomal entry site (IRES) and eGFP as selection marker. For production of lentivirus, subconfluent HEK 293 T cells were transfected with the *pgk:cryab* construct and the third generation packaging vectors pMDLg/pRPE, pRSV-rev and pMD2.G [13] by calcium phosphate precipitation. The medium was changed the next day and supernatant containing virus particles was collected 48 h later, filtered (0.45 μm pore size) and stored at −80 °C until usage. The concentration of infectious particles was determined by transducing HEK 293 T cells with serious dilutions of supernatants of the virus *pgk:cryab* or a control virus (*pgk:ev*) containing no cDNA and measurement of GFP expression 72 h later on a LSRII FACS instrument (BD, Franklin Lakes, NJ). HUVEC cells at passage 4 were seeded on gelatinized culture dishes 1 h prior to transduction with lentivirus containing supernatants at an MOI of 1. Polybrene (Sigma-Aldrich) was added to a final concentration of 5 μg/ml to improve transduction efficiency. Medium was exchanged after 12 h and cells were cultured for an additional 3 passages before sorting of GFP^+^ cells on a FACSVantage SE (BD). GFP expression was tested routinely by FACS during further culturing of the cells.

### In vitro stimulation of endothelial cells

Endothelial cells were rinsed with PBS and treated with recombinant mouse TNF-α (20 μg/ml, BioVision, Mountain View, CA) in EBM MV2 + 1 % FCS (for HUVEC) and DMEM (for MyEnd) for the indicated time periods.

### RNA extraction, cDNA synthesis and quantitative real-time PCR (qPCR)

RNA from adherently growing cells and cremaster muscles was extracted using the RNeasy Mini Kit (Qiagen, Hilden, Germany) or Micro Kit, respectively, with on-column DNase digestion, according to the manufacturer’s instructions. Between 500 ng and 1 μg of RNA were used for reverse transcription using random primers and SuperScript III reverse transcriptase (Life Technologies) according to the manufacturer’s instructions. Quantitative real-time PCR was done using Sybr-Green (Life Technologies), 0.25 μM forward and reverse primer and 0.5 μl cDNA per reaction, with human/mouse *hprt* serving as internal control. For primer sequences, please refer to table S1 in the supplement.

All reactions were performed in triplicates on an MX3005 instrument (Stratagene, Cedar Creek, TX). For gene expression analysis, relative expression values were calculated according to the formula: relative expression _gene x_ = 2^ − (Ct _gene x_ − Ct _internal reference_) and the mean expression and standard deviation for each triplicate was calculated.

### Western blot

Cells were washed with PBS and lysed with 1× LDS sample buffer and 1× sample reducing agent (Life Technologies). After homogenization by vigorous pipetting and incubation at 70 °C for 10 min, samples were separated on NuPage 4-12 % Bis–Tris Gels using MOPS buffer (Life Technologies) and transferred to Hybond-C extra (GE Healthcare, Chalfont St. Giles, UK) according to the manufacturer’s protocol. Membranes were blocked in 5 % non-fat dry milk in TBS + 0.01 % Tween (blocking solution) for 1 h. Primary antibodies diluted in blocking solution were incubated over night at 4 °C. Membranes were washed in TBS-T and incubated with HRP conjugated secondary antibodies. Membranes were washed several times in TBS-T before detection using ECL Prime substrate or CCD camera detection using Bio-Rad ChemiDoc™ MP Imaging System (Bio-Rad, Hercules, CA). Primary antibodies used were anti-αB-crystallin (Clone 1B6.1-3G4, Enzo LifeSciences, Farmingdale, NY), anti-Actin (sc-1615, Santa Cruz Biotech, Santa Cruz, CA), anti-β-catenin (610153, BD Biosciences), and anti-IκB (sc-371, Santa Cruz). Secondary antibodies were donkey anti-mouse-HRP (GE Healthcare), donkey anti-rabbit-HRP (GE Healthcare) and mouse anti-goat-HRP (Clone GT-34, Sigma-Aldrich).

### Nuclear translocation of p-p65

Human Umbilical Vein Endothelial Cells grown on coverslips were starved and activated as described above. At the indicated timepoints, cells were washed with TBS, fixed in zinc fix (0.1 M Tris–HCl, pH 7.5, 3 mM calcium acetate, 23 mM zinc acetate, and 37 mM zinc chiloride) with 0.2 % Triton ×-100 for 20 min at RT, washed, and blocked in 3 % FBS/TBS for 1 h at RT. Cells were stained with rabbit anti-pSer536-p65 (Cell Signaling, Danvers, MA), washed 3 times with TBS, incubated with donkey anti-rabbit-Alexa488 (Life Technologies), and counterstained with Hoechst33342 (2 μg/ml) before mounting. Images were taken on a Nikon Eclipse fluorescence microscope and analyzed using ImageJ (NIH, Bethesda, MD).

### FACS analysis

Endothelial cells were washed with PBS + 1 mM EDTA and gently detached using 0.01 % Trypsin in PBS + 1 mM EDTA. FACS buffer (1 % FCS, 0.02 % NaN_3_ in PBS) was added immediately once the cells became detached. Cells were incubated with primary antibodies diluted in FACS buffer for 1 h at 4 °C, washed with FACS buffer, and subsequently incubated with secondary antibodies for an additional 30 min. Directly before FACS analysis, cells were washed and resuspended in FACS buffer, and DAPI or PI (Sigma-Aldrich) were added to discriminate living and dead cells. Samples were analyzed on a LSRII cytometer (BD). The following primary antibodies were used (at 2 μg/ml): mouse control IgG_1_ (BD), mouse anti-human ICAM-1, mouse anti-human E-selectin, goat anti-mouse ICAM-1, goat anti-mouse VCAM-1 (all R&D Systems, Minneapolis, MN), mouse anti-human VCAM-1 (eBioscience, San Diego, CA) and PE labeled rat anti-mouse E-selectin (BD). The following secondary antibodies were used (10 μg/ml): goat anti-mouse IgG conjugated to Alexa488, goat anti-mouse IgG conjugated to Alexa555, and donkey anti-goat IgG conjugated to Alexa488 (all Life Technologies).

### Adhesion assay

Jurkat cells were labeled with the PKH26 red fluorescent cell linker kit (Sigma-Aldrich) according the manufacturer’s instructions and co-incubated with HUVEC monolayers in 24 well plates for 15 min at 37 °C on an orbital shaker to mimic flow. Subsequently, monolayers were washed 4 times with PBS and fixed in 4 % PFA before microscopic examination on an LSM700 inverted confocal microscope (Carl Zeiss, Jena, Germany). The number of adherent Jurkat cells per well was determined using ImageJ (NIH).

### Intravital microscopy

Male *cryab* -*/*- and wild type mice were anaesthetized with isoflurane (Abbott Laboratories, Abbott, IL) and the cremaster muscle was exposed for intravital microscopic observation of leukocytes as previously described [14]. An intravital microscope (Ortholux II, Leica Microsystems, Wetzlar, Germany) equipped with a 25′/0.6 W long distance water dipping objective (Leica Micorsystems) and a C3077 digital camera (Hamamatsu Photonics, Hamamatsu City, Japan) and an intravital microscope (DM5000 B, Leica Micorsystems) equipped with a 20×/0.5 W long distance water dipping objective and an ORCA C10600 digital camera (Hamamatsu Photonics) were used. For induction of inflammation, recombinant mouse TNF-α (500 μg/kg body weight, R&D Systems, in sterile saline) was injected intrascrotally 2.5 h prior to the surgical procedure. Single unbranched venules (35–50 μm in diameter) were chosen for observation throughout the experiment. 5 min long sequences were recorded for offline playback analysis at 3.5, 4.0 and 4.5 h after TNF-α administration. The flux of rolling cells was assumed as the average number of rolling leukocytes per min passing. Rolling velocity was measured for the first 10 neutrophils entering the field of view at each time point. Leukocytes were considered adherent if they remained stationary for at least 30 s, and total leukocyte adhesion was quantified as the number of adherent cells within a 100 μm length of venule. Leukocyte emigration was defined as the number of cells in the extravascular space within the field of view at the end of each 5 min sequence. Only cells adjacent to and clearly outside the venule were counted as emigrated.

### Statistical analysis

Statistical analysis was done using GraphPad Prism 5.0 (GraphPad Inc., La Jolla, CA). For comparison of two groups, student’s unpaired *t* test was used. For comparison of multiple groups, two-way ANOVA with Bonferroni post-test was used. A *p* value <0.05 was considered to be statistically significant.

## Results

### Ectopic expression of αB-crystallin in HUVEC increases TNF-α induced expression of E-selectin

**Fig. 1:**
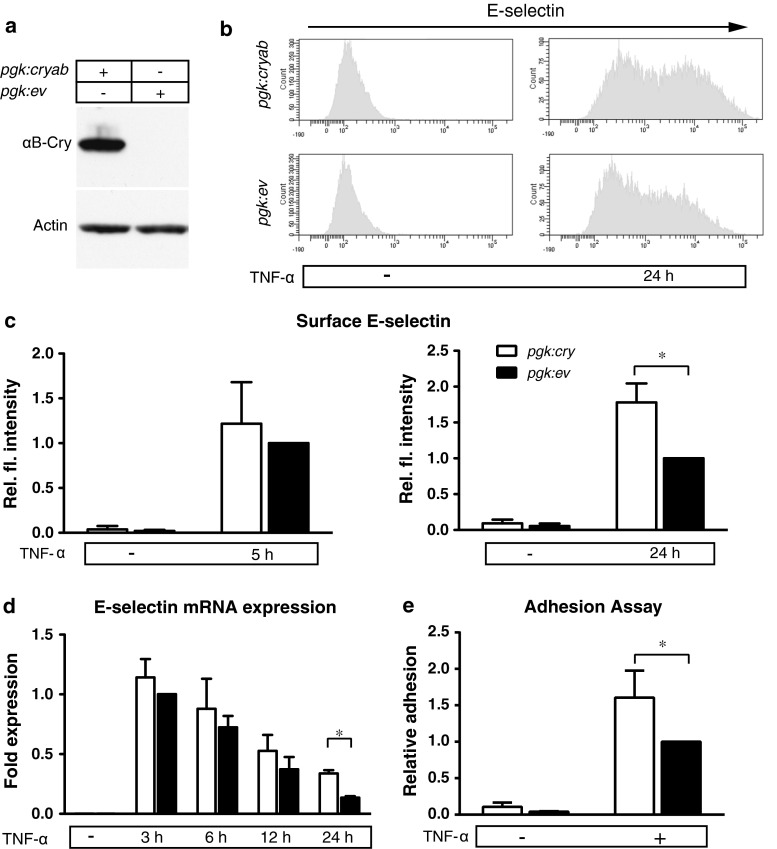
Ectopic expression of αB-crystallin increases E-selectin levels in HUVEC. **a** Representative Western Blot for αB-crystallin in HUVEC transduced with a lentiviral vector coding for full-length human αB-crystallin (*pgk:cryab*) or an empty control vector (*pgk:ev*). Actin served as loading control. **b** Representative FACS plots of E-selectin staining on HUVEC transduced with *pgk:cryab* and *pgk:ev* after treatment with TNF-α for 24 h. **c** Quantification of surface expression of E-selectin in HUVEC transduced with *pgk:cryab* (*white bars*) or empty vector controls (*black bars*). Cells were treated with TNF-α for 5 h (*left panel*) or 24 h (*right panel*) **d** HUVEC transduced with *pgk:cryab* (*white bars*) or empty vector controls (*black bars*) were stimulated for 3, 6, 12 and 24 h with TNF-α and expression of E-selectin relative to *hprt* was determined by qPCR (*Bars* represent mean ± SD fold expression compared to *pgk:ev* after 3 h of TNF-α (normalized data from 3 independent experiments), * = *p* < 0.05). **e** HUVEC transduced with *pgk:cryab* (*white bars*) or empty vector controls (*black bars*) were grown to confluency and stimulated with TNF-α for 24 h before co-incubation with Jurkat cells for 15 min. Firmly adherent cells were microscopically quantified. (*Bars* represent mean ± SD (normalized data from 3 individual independent), * = *p* < 0.05)

To analyze the role of αB-crystallin in endothelial activation, we initially used human umbilical vein endothelial cells that essentially do not express αB-crystallin under basal conditions. Using lentiviral transduction we created HUVEC stably expressing αB-crystallin under control of the *pgk* promotor. In these cells (*pgk:cryab*), αB-crystallin is readily detectable by western blot, while no αB-crystallin is detected in cells transduced with an empty control vector (*pgk:ev*) (Fig. 1a). Overexpression of αB-crystallin in HUVEC had no significant effects on cell proliferation (Fig S1a) To determine if αB-crystallin expression affects expression of endothelial adhesion molecules, we treated *pgk:cryab* and *pgk:ev* HUVEC with TNF-α and analyzed surface expression of ICAM-1, VCAM-1 and E-selectin by FACS. Notably, we found a clear increase in mean fluorescent intensity of surface E-selectin staining on *pgk:cryab* compared to *pgk:ev* HUVEC after 24 h but not 5 h of TNF-α stimulation, while surface levels of ICAM-1 and VCAM-1 were similar at both time points (Fig. 1b, c and Fig S1b-c). Accordingly, the percentage of E-selectin positive cells was significantly higher after 24 h of TNF-α treatment in *pgk:cryab* HUVEC (58 %) as compared to *pgk:ev* HUVEC (44 %). Western blot analysis revealed increased levels of E-selectin in *pgk:cryab* HUVEC lysates, indicating that the αB-crystallin-induced increase in surface expression was associated with increased protein levels (Fig S1d-e). Consistent with this, gene expression analysis by quantitative real-time PCR (qPCR) revealed an increase in TNF-α-induced mRNA expression of E-selectin after 24 h of stimulation in *pgk:cryab* HUVEC as compared to control cells (Fig. 1d). Collectively, this data suggests that ectopic expression of αB-crystallin enhances E-selectin levels mainly through increasing TNF-α-induced transcriptional activation of gene expression.

### Ectopic expression of αB-crystallin in HUVEC leads to enhanced leukocyte adhesion to endothelium

To assess the functional impact of αB-crystallin associated increase in surface E-selectin in HUVEC, we analyzed leukocyte adhesion to endothelial cells in vitro. To measure leukocyte adhesion, monolayers of *pgk:cryab* and *pgk:ev* HUVEC were stimulated with TNF-α for 24 h. Subsequently we added fluorescently labeled Jurkat cells, a lymphoblastic cell line which expresses receptors for ICAM-1, VCAM-1 and ligands for E-selectin, and incubated them for 15 min on an orbital shaker to simulate flow. Firm adhesion of Jurkat cells was determined by microscopy after extensive washing. Under these conditions, an increased number of Jurkat cells adhered to *pgk:cryab* HUVEC as compared to *pgk:ev* HUVEC (Fig. 1e). This suggests that αB-crystallin enhances capture of leukocytes, consistent with its role in up-regulating E-selectin.

### Decreased TNF-α-induced expression of endothelial adhesion molecules in αB-crystallin-deficient microvascular endothelial cells

**Fig. 2:**
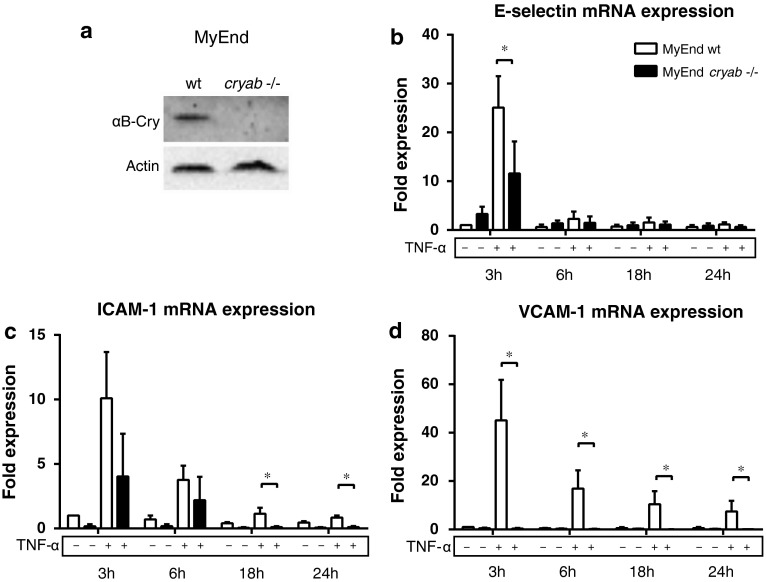
TNF-α-induced endothelial activation is reduced in endothelial cells derived from *cryab* -/- mice. **a** Representative western blot showing αB-crystallin expression in MyEnd wild type and MyEnd *cryab* -*/*- cells. **b**, **c**, **d** MyEnd wild type and MyEnd *cryab* -*/*- cells were treated with TNF-α for 3, 6, 18 and 24 h and expression of E-selectin (**b**), ICAM-1 (**c**) and VCAM-1 (**d**) relative to *hprt* was determined by qPCR. (*Bars* represent mean ± SD (normalized data from 3 independent experiments), * = *p* < 0.05)

To investigate the role of endogenous αB-crystallin in inflammatory activation of endothelial cells, we used endothelial cell lines established from myocardial microvascular endothelial cells isolated from 129S6 wild type mice (MyEnd wt) or αB-crystallin-deficient (*cryab* -*/*-*)* mice (MyEnd *cryab* -*/*-) [2]. As expected, MyEnd wt endothelial cells expressed robust levels of αB-crystallin while no αB-crystallin could be detected in MyEnd *cryab* -*/*- cells (Fig. 2a). To analyze if αB-crystallin deficiency affected endothelial activation, cells were treated with TNF-α and mRNA levels of E-selectin, ICAM-1 and VCAM-1 were measured by qPCR. We noticed a striking ~50 % reduction in E-selectin mRNA levels in MyEnd *cryab* -*/*- cells as compared to MyEnd wt after 3 h of TNF-α stimulation, and differences in expression were maintained up to 24 h (Fig. 2b). Interestingly, αB-crystallin deficiency in MyEnd cells also decreased TNF-α-induced mRNA expression of ICAM-1 and VCAM-1 (Fig. 2c, d). In line with this, surface expression of E-selectin, ICAM-1 and VCAM-1 were reduced in *cryab* -/- cells after 5 h of TNF-α treatment (Fig S2a-c). We conclude that αB-crystallin deficiency is associated with reduced TNF-α-induced mRNA expression of E-selectin, ICAM-1 and VCAM-1, while ectopic expression of αB-crystallin only affects E-selectin expression in our experimental set-up.

### TNF-α-induced NF-κB activation is reduced in the absence of αB-crystallin

**Fig. 3:**
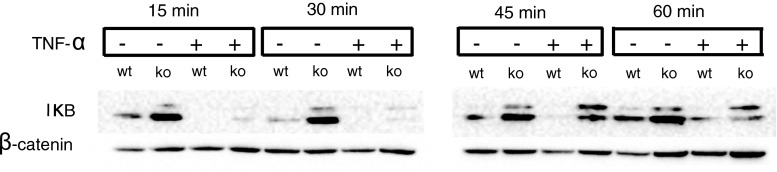
TNF-α-induced activation of NF-κB is reduced in the absence of αB-crystallin. MyEnd wt and MyEnd *cryab* -*/*- cells were treated with TNF-α for 15, 30, 45 min and 1 h, protein lysates were prepared and expression of IκB was determined by western blot. One representative blot of three independent experiments is shown

Since αB-crystallin has been implicated in both positive and negative regulation of the NF-κB-pathway in different cell types [15, 16], we investigated if αB-crystallin modulates NF-κB activation in endothelial cells. Through western blot analysis of TNF-α-stimulated MyEnd *cryab* -*/*- and MyEnd wt cells, we found elevated levels and reduced degradation of IκB in αB-crystallin-deficient endothelial cells (Fig. 3). Furthermore, nuclear translocation of phospho-NF-κB p65 was impaired in *cryab* -/- MyEnd cells (Fig S2c,d). This demonstrates that absence of αB-crystallin leads to increased IκB expression and inhibition of NF-κB mediated activation of endothelial cells, resulting in reduced expression of NF-κB target genes ICAM-1, VCAM-1 and E-selectin in MyEnd *cryab* -*/*- cells.

### Leukocyte–endothelial interactions are altered in αB-crystallin deficient (*cryab* -*/*-) mice

**Fig. 4:**
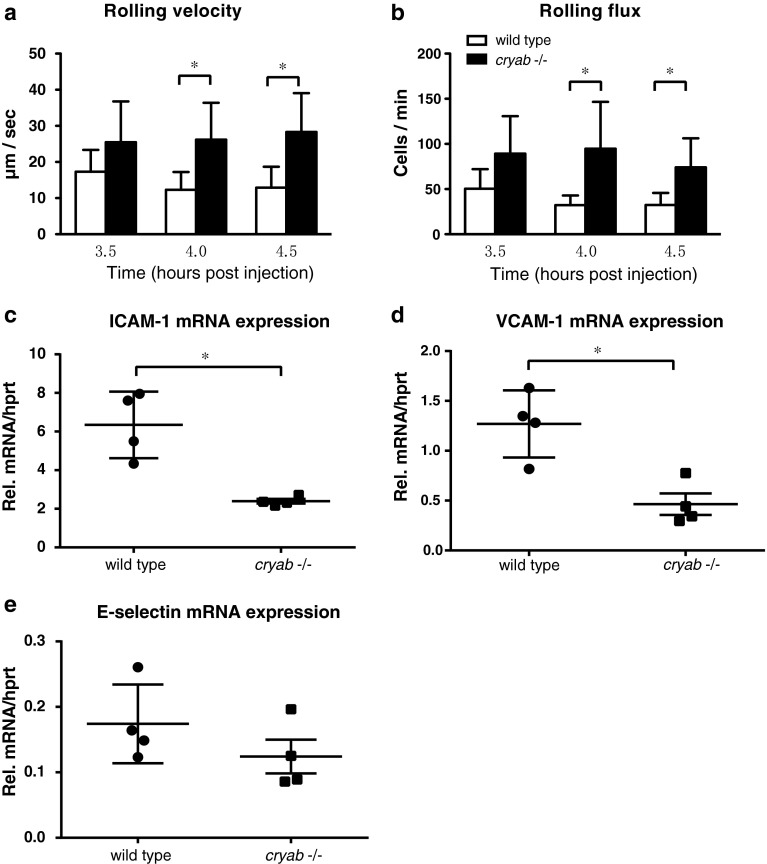
In vivo leukocyte–endothelial interactions are altered in *cryab* -*/*- mice, associated with decreased expression of endothelial adhesion molecules. Intravital microscopy was used to analyze leukocyte–endothelial interactions in inflamed venules of the cremaster muscle of wild type (*white bars*) and *cryab* -*/*- mice (*black bars*). Analysis of rolling velocity (**a**) and rolling flux (**b**) 3.5, 4, and 4.5 h after intrascrotal injection of TNF-α (n = 5, mean ± SD, *p < 0.05). qPCR analysis of mRNA expression of ICAM-1 (**c**), VCAM-1 (**d**) and E-selectin (**e**) relative to *hprt* in mouse cremaster muscles harvested 4.5 h after intrascrotal injection of TNF-α (n = 4, mean ± SD, *p < 0.05)

To test whether αB-crystallin dependent modulation of endothelial adhesion molecules affects leukocyte–endothelial interactions in vivo, we used intravital microscopy to study leukocyte rolling, adhesion and emigration in inflamed cremaster venules in *cryab* -*/*- and wild type mice. A single intrascrotal injection of TNF-α was used to induce endothelial activation. In line with reduced expression of E-selectin, ICAM-1 and VCAM-1, we found that leukocyte rolling velocity was significantly higher in *cryab* -*/*- mice than in wild type mice, 4 and 4.5 h after injection of TNF-α (Fig. 4a). We also observed a higher total number of rolling leukocytes in *cryab* -*/*- mice (rolling flux, Fig. 4b), while the number of firmly adherent leukocytes and emigrated leukocytes was not significantly altered (Fig S3 a-b). This was associated with decreased mRNA levels of ICAM-1 and VCAM-1 in TNF-α treated *cryab* -*/*- cremaster muscles as analyzed by qPCR (Fig. 4c, d), while CD31 mRNA levels were unchanged (Fig S3c). Vascular expression of ICAM-1 and VCAM-1 was confirmed by immunofluorescence staining (Fig S3d). A trend towards lower E-selectin mRNA expression in *cryab* -*/*- cremaster muscles was also noted, but did not reach statistical significance (Fig. 4e). Taken together, these results are consistent with a role for αB-crystallin in regulating endothelial–leukocyte interactions by increasing adhesion molecule expression on activated endothelium in vivo.

## Discussion

αB-crystallin has been attributed diverse cellular functions, including cytoskeletal stabilization, regulation of reactive oxygen species, and potent anti-apoptotic activity. Various types of endothelial cells, including human dermal microvascular endothelial cells (HDMEC) and bovine capillary endothelial cells (BCE), express αB-crystallin in culture, while expression is not detectable in e.g. HUVEC. αB-crystallin expression is up-regulated in tumor associated blood vessels [1, 17], but the vascular expression pattern of αB-crystallin in different organs, vascular beds, and different types of pathologies has not been thoroughly investigated. We have previously shown that αB-crystallin is up-regulated during VEGF-A-induced tubular morphogenesis and promotes angiogenesis by inhibiting caspase-3 activation in endothelial cells, thus increasing cell survival [1]. Subsequently, Kase et al. demonstrated an important role of αB-crystallin in increasing stability and secretion of VEGF during physiological angiogenesis [5]. Here, we describe an additional, formerly unappreciated function of αB-crystallin in regulating adhesion molecules during endothelial activation.

In vitro, we found that αB-crystallin enhances TNF-α induced NF-κB activation and expression of adhesion molecules in endothelial cells. Ectopic expression of αB-crystallin in HUVEC resulted in increased E-selectin expression and leukocyte adhesion in response to TNF-α. Both protein and mRNA levels of E-selectin were increased, indicating that αB-crystallin regulates E-selectin expression on the transcriptional level. Correspondingly, TNF-α induced expression of adhesion molecules ICAM-1, VCAM-1, and E-selectin was reduced in αB-crystallin deficient endothelial cells. In line with these findings, a decrease in TNF-α induced mRNA expression of ICAM-1, VCAM-1 and E-selectin was noted in αB-crystallin-deficient mice. αB-crystallin has been suggested to affect the NF-κB signaling pathway, both positively and negatively, in different systems [15, 16]. Notably, Adhikari et al. [15] recently showed that αB-crystallin expression increases IKKβ activity, thereby promoting phosphorylation and degradation of IκB in response to TNF-α and, correspondingly, activation of NF-κB in the myoblastic cell line C2C12. In agreement to this, we found that IκB levels were increased in αB-crystallin-deficient endothelial cells under basal conditions, and that TNF-α induced degradation of IκB and nuclear accumulation of phospho-NF-κB p65 was inhibited if αB-crystallin was absent. This indicates that αB-crystallin exerts a similar function in endothelial cells as in myoblasts, i.e. to regulate NF-κB activation by promoting degradation of IκB, possibly by interacting with and stimulating IKKβ. A difference in IκB degradation however could not be observed in *pgk:cryab* transduced HUVECs (data not shown), suggesting that αB-crystallin may have additional effects on E-selectin expression.

Using intravital microscopy, we found that leukocyte rolling velocity in TNF-α activated venules is strikingly higher in αB-crystallin deficient (*cryab* -*/*-) mice. This finding is in line with various studies using genetic mouse models demonstrating that deficiency in ICAM-1, E-selectin or E-selectin ligands results in higher leukocyte rolling speed in vivo [18–20]. We also observed a higher number of rolling leukocytes (flux) in *cryab* -*/*- mice, indicating a lower grade of activation. However, the numbers of adherent and emigrated leukocytes were not significantly different between groups due to great variations between individuals. Furthermore, we have shown previously that unchallenged *cryab* -*/*- mice have normal numbers of circulating leukocytes, indicating that the increased leukocyte flux is not due to a higher leukocyte number in these mice [7]. Possibly, compensatory mechanisms, for example an increased surface exposure of P-selectin or other endothelial adhesion molecules, might also be involved. Nevertheless, lack of αB-crystallin is clearly associated with altered leukocyte–endothelial interactions in inflamed venules in vivo. This suggests that endothelial αB-crystallin may act as a positive regulator of leukocyte recruitment in inflammatory conditions, particularly where its expression is induced by e.g. angiogenic or stress-related signals. Further studies are warranted to test this hypothesis in vivo.

## Electronic supplementary material

Below is the link to the electronic supplementary material.

Supplementary material 1 (DOCX 23 kb)

Supplementary material 2 (PDF 7481 kb)
