# Supplementary material for: αB-crystallin/HspB5 regulates endothelial–leukocyte interactions by enhancing NF-κB-induced up-regulation of adhesion molecules ICAM-1, VCAM-1 and E-selectin
Source: Angiogenesis. 2013 Aug 9;16(4):975–83. doi: 10.1007/s10456-013-9367-4 (PMC3779083; doi:10.1007/s10456-013-9367-4)
Supplement: Supplementary file 1 — Supplementary material 1 (DOCX 23 kb) [file 10456_2013_9367_MOESM1_ESM.docx]

**SUPPLEMENTARY FIGURE LEGENDS**

Fig. S1 Total E-selectin levels are increased, but ICAM-1 and VCAM-1 levels **are unchanged in αB-crystallin-expressing HUVEC**

a) Proliferation of HUVEC cells transduced with *pgk:cryab* or *pgk:ev* was determined by cell counting over a period of 96 h after plating. Bars represent mean proliferation (in % of plated cells at day 0, two independent experiments). b-c) FACS analysis of ICAM-1 (b) and VCAM-1 (c) expression in HUVEC transduced with *pgk:cryab* (white bars) or *pgk:ev* (black bars) stimulated with TNF-α for the indicated time periods (Bars represent mean ± SD (normalized data from 4 independent experiments)). d) Representative western blot analysis of E-selectin protein expression in HUVEC transduced with *pgk:cryab* or *pgk:ev* after 5h and 24h of TNF-α stimulation. e) Quantification of pixel intensity in western blot analysis of TNF-α-induced E-selectin expression in HUVEC transduced with *pgk:cryab* (white bars) or *pgk:ev* (black bars). Bars show E-selectin protein expression normalized to actin (Mean ± SD (normalized data from 3 independent experiments), * = p < 0.05)

**Fig. S2 FACS analysis of E-Selectin, ICAM-1 and VCAM-1 expression and nuclear translocation of p-p65 in TNF-α treated MyEND cells.**

MyEND cells were activated with TNF-α for 5h. Expression of E-selectin (a), ICAM-1 (b) and VCAM-1 (c) was determined by FACS (Bars represent mean ± SD (normalized data from 2-3 independent experiments)) d) Representative images of p-p65 staining (red) in wild type (left panels) and *cryab* -/- (right panels) MyEnd cells stimulated with TNF-α for the indicated time periods e) Quantification of nuclear p-p65 staining intensity in MyEnd cells (Mean ± SD of at least 50 nuclei / condition, * p < 0.05 (two-way ANOVA with bonferroni post-test))

**Fig. S3 Leukocyte adhesion and emigration and vascularity in cremaster muscles are not altered in *cryab^-/-^* mice**

Analysis of number of adherent cells (a) and emigrated cells (b) by intravital microscopy, 3.5 h, 4 h, and 4.5 h after intrascrotal injection of TNF-α (n = 5, mean ± SD). (c) qPCR analysis of mRNA expression of CD31 relative to *hprt* in mouse cremaster muscles 4.5h after injection of TNF-α (n = 4, mean ± SD). d) Representative microscopic images of VCAM-1 and ICAM-1 (green) in CD31+ (red) vessels in tissue sections from cremaster muscles, 4.5h after injection of TNF-α
