# Supplementary material for: αB-crystallin/HspB5 regulates endothelial–leukocyte interactions by enhancing NF-κB-induced up-regulation of adhesion molecules ICAM-1, VCAM-1 and E-selectin
Source: Angiogenesis. 2013 Aug 9;16(4):975–83. doi: 10.1007/s10456-013-9367-4 (PMC3779083; doi:10.1007/s10456-013-9367-4)
Supplement: Supplementary file 2 — Supplementary material 2 (PDF 7481 kb) [file 10456_2013_9367_MOESM2_ESM.pdf]

αB-Crystallin/HspB5 regulates endothelial-leukocyte interactions by enhancing NF-κB-induced up-regulation of adhesion molecules ICAM-1, VCAM-1 and E-selectin

Lothar C. Dieterich[1, 4, 5], Hua Huang[1, 4], Sara Massena[2], Nikola Golenhofen[3], Mia Phillipson[2], Anna Dimberg[1,*]

[1] Department of Immunology, Genetics and Pathology, The Rudbeck Laboratory, Uppsala University, SE-751 85 Uppsala, Sweden

[2] Department of Medical Cell Biology, Uppsala University, SE-751 23 Uppsala, Sweden

[3] Institute of Anatomy and Cell Biology, University of Ulm, Albert-Einstein-Allee 11, 89081, Ulm, Germany

[4] LCD and HH contributed equally to this manuscript

[5] Current address: Institute of Pharmaceutical Sciences, Swiss Federal Institute of Technology (ETH) Zurich, CH-8093 Zurich, Switzerland

* Corresponding author: Anna Dimberg, Department of Immunology, Genetics and Pathology, Rudbeck Laboratory, Uppsala University, SE-751 85 Uppsala, Sweden. Phone: +46 18 471 4636 Fax: +46 18 55 89 31 Email: Anna.Dimberg@igp.uu.se

SUPPLEMENTARY FIGURES S1, S2, S3 AND TABLE S1

Fig. S1

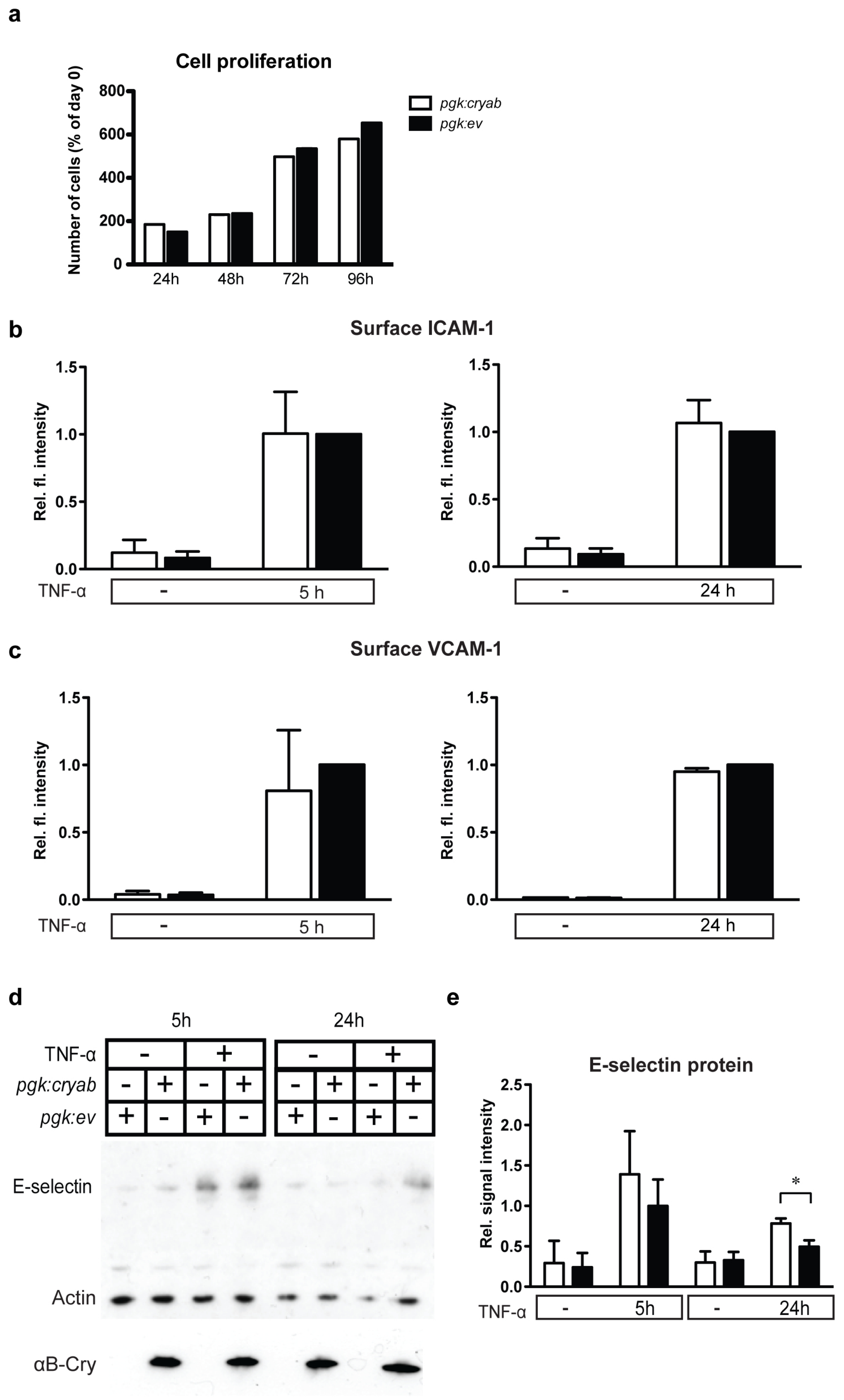

a
Cell proliferation
Number of cells (% of day 0)
800
600
400
200
0
24h
48h
72h
96h
pgk:cryab
pgk:ev
b
Surface ICAM-1
Rel. fl. intensity
1.5
1.0
0.5
0.0
TNF-α
-
5 h
24 h
c
Surface VCAM-1
Rel. fl. intensity
TNF-α
-
5 h
24 h
d
5h
24h
TNF-α
pgk:cryab
pgk:ev
E-selectin
Actin
αB-Cry
e
E-selectin protein
Rel. signal intensity
2.5
2.0
1.5
1.0
0.5
0.0
*
TNF-α
-
5h
24h

Fig. S2

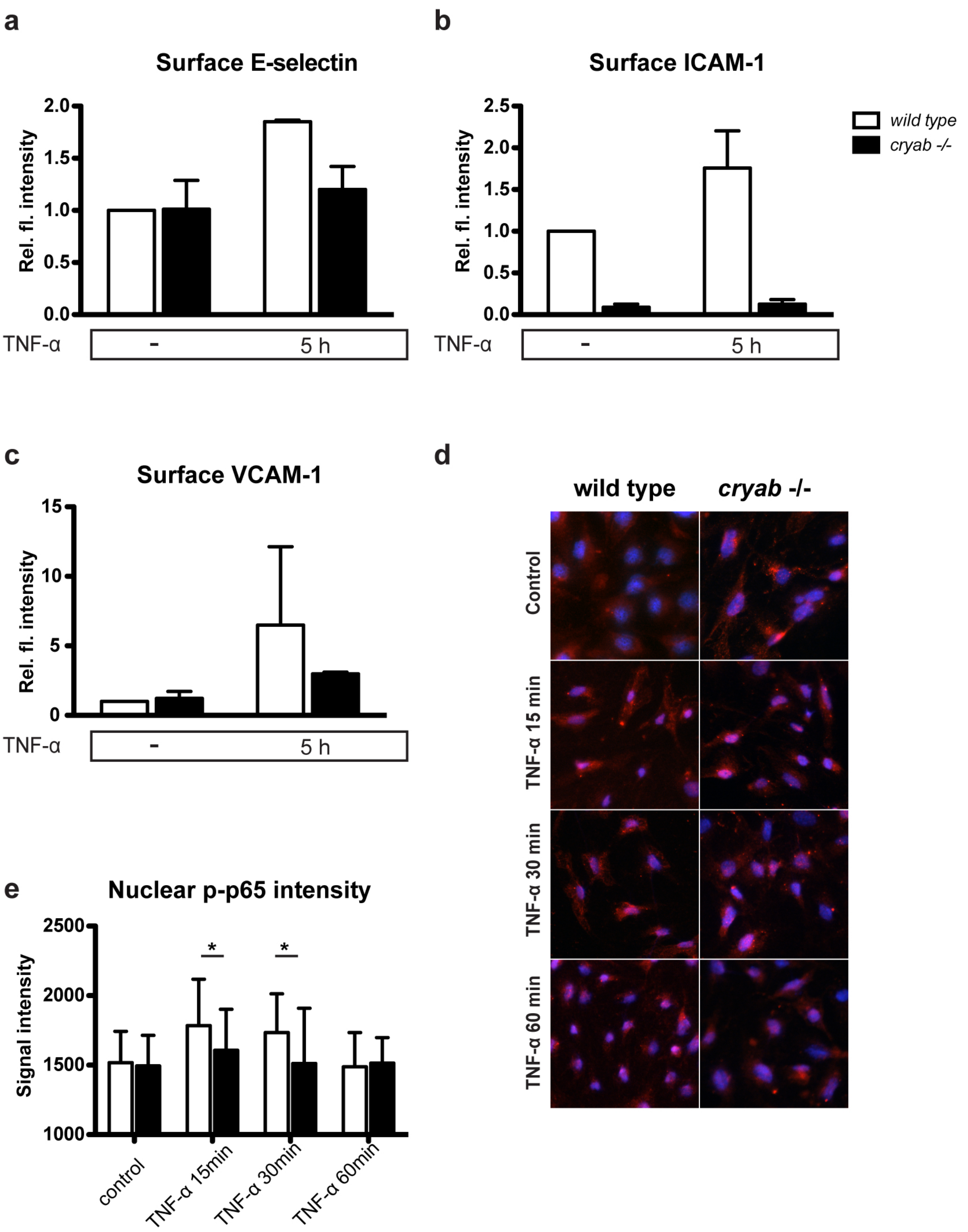
a
Surface E-selectin
Rel. fl. intensity
2.0
1.5
1.0
0.5
0.0
TNF-α
-
5 h
b
Surface ICAM-1
Rel. fl. intensity
2.5
2.0
1.5
1.0
0.5
0.0
TNF-α
-
5 h
wild type
cryab -/-
c
Surface VCAM-1
Rel. fl. intensity
15
10
5
0
TNF-α
-
5 h
d
wild type
cryab -/-
Control
TNF-α 15 min
TNF-α 30 min
TNF-α 60 min
e
Nuclear p-p65 intensity
Signal intensity
2500
2000
1500
1000
*
*
control
TNF-α 15min
TNF-α 30min
TNF-α 60min

Fig. S3

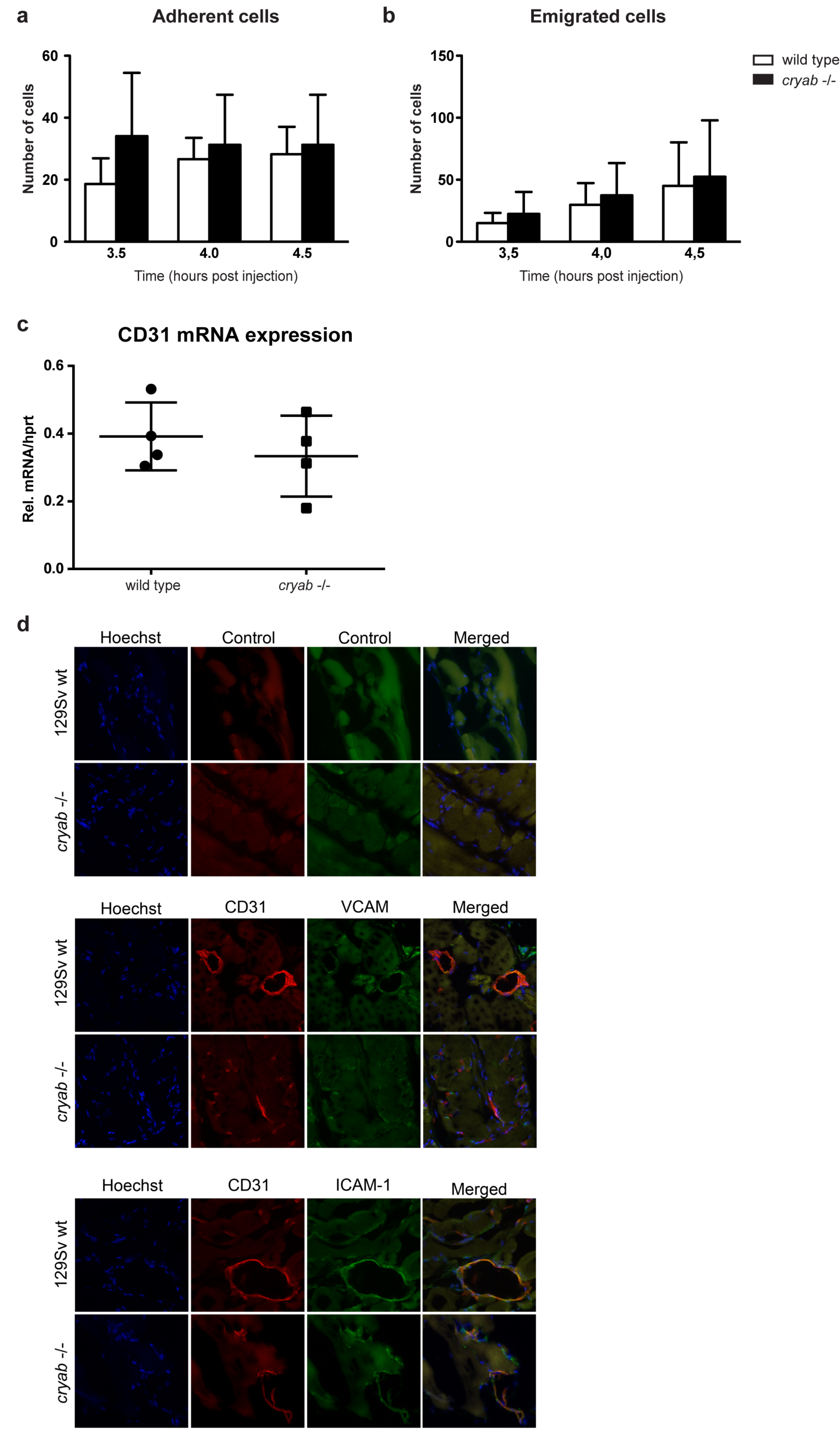
a
Adherent cells
Number of cells
60
40
20
0
3.5
4.0
4.5
Time (hours post injection)
b
Emigrated cells
Number of cells
150
100
50
0
3,5
4,0
4,5
Time (hours post injection)
wild type
cryab -/-
c
CD31 mRNA expression
Rel. mRNA/hprt
0.6
0.4
0.2
0.0
wild type
cryab -/-
d
Hoechst
Control
Control
Merged
129Sv wt
cryab -/-
Hoechst
CD31
VCAM
Merged
129Sv wt
cryab -/-
Hoechst
CD31
ICAM-1
Merged
129Sv wt
cryab -/-

Supplementary Table 1: qPCR primer sequences

	Forward primer (5’-3’)	Reverse primer (5’-3’)
mouse hprt	CAAACTTTGCTTTCCCTGGT	TCGAGAGGTCCTTTTCACC
mouse pecam	TACTGCAGGCATCGGCAAA	GCATTTCGCACACCTGGAT
mouse ve-cadherin	AGGACAGCAACTTCACCCTCA	AACTGCCCATACTTGACCGTG
mouse icam	CCGCTACCATCACCGTGTA	CAGAGGTCTCAGCTCCACACT
mouse vcam	TGGGAAGCTGGAACGAAGTA	CTCTGGATCCTTGGGGAAA
mouse sele	GCGCTTTCTCTCTGCTCTTG	ATGAGCTCACTGGAGGCATT
human hprt	CTTTGCTGACCTGCTGGATT	TCCCCTGTTGACTGGTCATT
human cryab	TGATTGAGGTGCATGGAAAA	GTGGAACTCCCTGGAGATGA
human icam	GCCAACCAATGTGCTATTCA	GTTCCACCCGTTCTGGAGT
human vcam	CCACAGTAAGGCAGGCTGTA	TTCTTGCAGCTTTGTGGATG
human sele	AGTGTGACCCTGGCTTCAGT	CTTCCATGCTCAGGGGATT
